# Human adolescent brain similarity development is different for paralimbic versus neocortical zones

**DOI:** 10.1073/pnas.2314074121

**Published:** 2024-08-09

**Authors:** Lena Dorfschmidt, František Váša, Simon R. White, Rafael Romero-García, Manfred G. Kitzbichler, Aaron Alexander-Bloch, Matthew Cieslak, Kahini Mehta, Theodore D. Satterthwaite, The NSPN Consortium, Richard A. I. Bethlehem, Jakob Seidlitz, Petra E. Vértes, Edward T. Bullmore

**Affiliations:** ^a^Department of Psychiatry, University of Cambridge, Cambridge CB2 0SZ, United Kingdom; ^b^Department of Psychiatry, University of Pennsylvania, Philadelphia, PA 19104; ^c^Lifespan Brain Institute, The Children’s Hospital of Philadelphia and Penn Medicine, Philadelphia, PA 19139; ^d^Department of Neuroimaging, King’s College London, London SE5 8AF, United Kingdom; ^e^Instituto de Biomedicina de Sevilla Hospital Universitario Virgen del Rocio/Consejo Superior de Investigaciones Científicas Universidad de Sevilla, Centro de Investigación Biomédica En Red Salud Mental, Instituto de Salud Carlos, Dpto. de Fisiología Médica y Biofísica, Sevilla 41013, Spain; ^f^Department of Clinical Neurosciences and Cambridge University Hospitals National Health Service Trust, University of Cambridge, Cambridge CB2 2PY, United Kingdom; ^g^Department of Child and Adolescent Psychiatry and Behavioral Sciences, Children’s Hospital of Philadelphia, Philadelphia, PA 19139; ^h^Penn Lifespan Informatics and Neuroimaging Center, Perelman School of Medicine, University of Pennsylvania, Philadelphia, PA 19104; ^i^Department of Psychology, University of Cambridge, Cambridge CB2 3EB, United Kingdom

**Keywords:** connectome, neuroimaging, isocortex, graph, adolescence centrality

## Abstract

Adolescent development of human brain structural and functional networks is increasingly recognized as fundamental to emergence of typical and atypical adult cognitive and emotional proodal magnetic resonance imaging (MRI) data collected from N ∼ 300 healthy adolescents (51%; female; 14 to 26 y) each scanned repeatedly in an accelerated longitudinal design, to provide an analyzable dataset of 469 structural scans and 448 functional MRI scans. We estimated the morphometric similarity between each possible pair of 358 cortical areas on a feature vector comprising six macro- and microstructural MRI metrics, resulting in a morphometric similarity network (MSN) for each scan. Over the course of adolescence, we found that morphometric similarity increased in paralimbic cortical areas, e.g., insula and cingulate cortex, but generally decreased in neocortical areas, and these results were replicated in an independent developmental MRI cohort (N ∼ 304). Increasing hubness of paralimbic nodes in MSNs was associated with increased strength of coupling between their morphometric similarity and functional connectivity. Decreasing hubness of neocortical nodes in MSNs was associated with reduced strength of structure–function coupling and increasingly diverse functional connections in the corresponding fMRI networks. Neocortical areas became more structurally differentiated and more functionally integrative in a metabolically expensive process linked to cortical thinning and myelination, whereas paralimbic areas specialized for affective and interoceptive functions became less differentiated, as hypothetically predicted by a developmental transition from periallocortical to proisocortical organization of the cortex. Cytoarchitectonically distinct zones of the human cortex undergo distinct neurodevelopmental programs during typical adolescence.

## Significance

We used MRI to investigate the adolescent development of human brain networks. In each of about 500 brain scans, we estimated the morphometric similarity, i.e. the similarity in terms of multiple structural magnetic resonance imaging (MRI) metrics, and the functional MRI connectivity, between each possible pair of 358 brain regions. During adolescence, morphometric similarity increased in paralimbic areas (which became more hub-like) and decreased in other cortical areas (which became more dissimilar or differentiated). Paralimbic areas also had distinct developmental trajectories of structure–function coupling and integrative connectivity of functional networks. Adolescent brain network development is different for paralimbic areas, specialized for linking cognitive, visceral, and emotional states, compared to neocortical areas specialized for sensorimotor and “higher-order” cognitive processes.

Magnetic resonance imaging (MRI) studies of human brain structure during adolescence and childhood have already identified two major developmental processes that are on going during the maturational transition from birth to adult brain organization: i) after peaking in early childhood, cortical gray matter volume and thickness monotonically decrease during adolescence; while ii) protracted myelination of the cortex sees peak white matter volumes reached in early adulthood (1). Anatomical MRI maps of cortical thinning and myelination markers like magnetization transfer (MT) are highly (negatively) correlated, indicating that these may be technically and/or biologically confounded measurements of the same underlying process of synaptic pruning and consolidation (2, 3). These and other large-scale, long-term neurodevelopmental programs are thought to be fundamental to the emergence of adult cognitive functions and social behaviors (4–6).

To date, neurodevelopmental MRI data have largely been studied using models of change in brain structure measured globally, or one region at a time (5, 7, 8). It is now timely to understand more about developmental change in brain structure measured by network or connectomic metrics, given the dominant known adolescent process of pruning and consolidation of (synaptic) connectivity between neurons and regions. A technical challenge for developmental network neuroscience has been the estimation of anatomical or structural connectivity between hundreds of regional (cortical and subcortical) nodes in each individual’s brain scan. Previous studies have used structural covariance analysis of a single MRI metric measured at each region in a large group of scans (2, 4); or tractography algorithms to reconstruct the fascicles and tracts of white matter projections between cortical areas (9). However, both approaches are problematic in different ways. Structural covariance analysis does not resolve the network organization of an individual scan, which is obviously crucial for within-subject developmental modeling of repeated measures in a longitudinal design. Tractography networks can be reconstructed from an individual’s diffusion-weighted imaging (DWI) data; but the resulting connectomes generally suffer from underestimation of long-range connections, e.g., between bilaterally symmetric cortical areas (10–12), and have been unfavorably benchmarked against structural covariance and newer methods for estimating the anatomical connectome in a single scan (13, 14).

An alternative approach has emerged recently based on the concept that cytoarchitectonically similar regions are more likely to be axonally connected, as articulated in more detail by the structural model of Barbas and colleagues (15, 16). On this basis, estimating the similarity between cortical or subcortical areas, each structurally phenotyped by one or more MRI metrics, could provide a tractable and plausible proxy for their axonal interconnectivity (17). This principle of homophily—“like connects with like”—has been demonstrated in nervous systems across scales and species. At the microscopic (cellular) level, synaptic connections are more likely to form between two functionally and developmentally similar neurons; at the macroscopic (whole brain) level, animal models have shown that there is greater interareal connectivity via large-scale axonal tracts between regions that have similar laminar structure and cellular composition (18). Last, generative modeling approaches have shown that a simple two-parameter model of distance-penalized homophily (19, 20) simulates many aspects of human brain network organization as an economic trade-off between wiring cost and topological complexity (21).

**Fig. 1.**
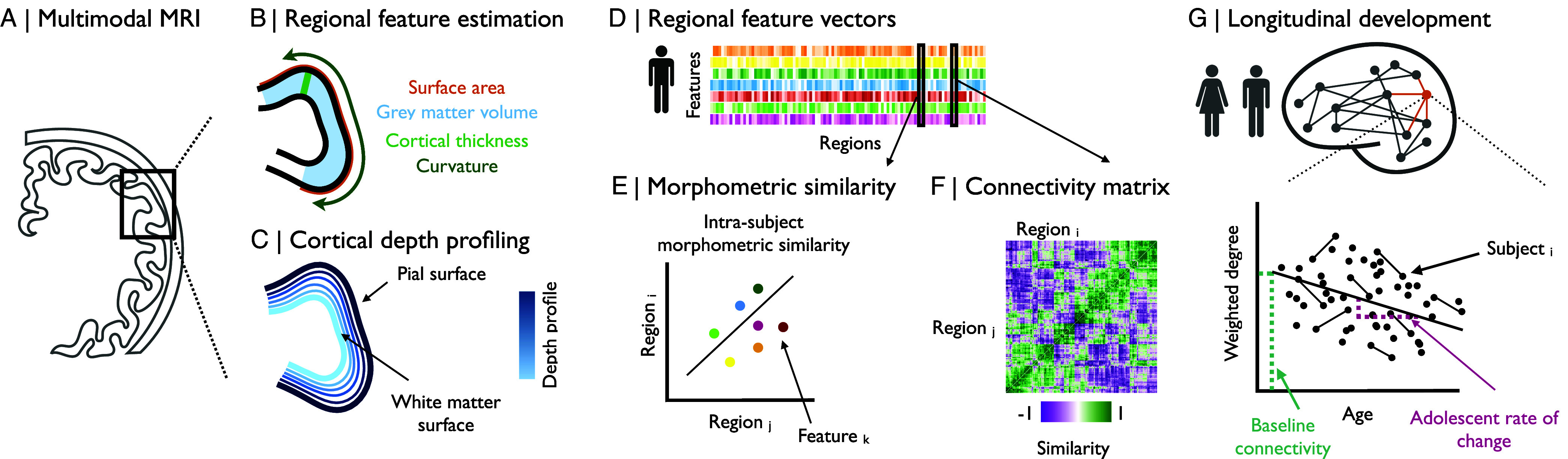
Modeling of adolescent changes in morphometric similarity. We estimated morphometric similarity networks (MSNs) from multimodal MRI brain scanning data. (*A*) These images were parcellated into 358 predefined cortical regions. (*B*) Macrostructural MRI phenotypes, like CT, GM, and SA, were estimated for each cortical area overall. (*C*) Additionally, depth-dependent profiling was used to construct multiple cortical surfaces between the white matter surface and the pial surface for estimation of MT, a microstructural MRI phenotype, with fine-grained laminar resolution at 70% of cortical depth. (*D*) We constructed a feature matrix of multiple features estimated at each region for each individual subject, resulting in a subject-specific {Regions×Features} multimodal MRI data matrix. (*E*) We estimated the similarity between each pair of cortical areas in terms of the pairwise correlations between regional feature vectors comprising multiple normalized macro- and microstructural MRI features estimated at each region. (*F*) We compiled all possible interareal similarity measures in a subject-specific {Regions×Regions} association matrix or morphometric similarity network. (*G*) We estimated adolescence-related changes in weighted degree, i.e., the mean weight over all of each node’s edges: the baseline functional connectivity, as the predicted nodal degree at age 14, and the rate of change of hubness, as the slope of a linear regression of age on weighted degree.

Morphometric similarity translates this invasive or in silico work to in vivo human brain networks by estimating all pair-wise interregional correlations of a multimodal MRI feature vector measured at each region. morphometric similarity network (MSN) can be built from any combination of structural MRI metrics (Fig. 1*A*), including i) macrostructural metrics, like cortical thickness (CT), gray matter volume (GM), and surface area (SA), which aggregate data from multiple voxels representing an anatomical region to estimate its geometric properties on the centimeter scale; and ii) microstructural metrics, which are representative of some aspect of brain tissue composition on the millimeter scale of a single voxel, e.g., MT is widely regarded as a proxy for cortical myelination (Fig. 1 *B* and *C*). MSNs have been shown to correlate with the “gold standard” of anatomical connectivity, axonal tract tracing data, in the macaque monkey (13, 17), thus validating morphometric similarity as a proxy for axonal connectivity. Network phenotypes, like the degree of connectivity or “hubness” of each regional node in the connectome, have also been used to predict individual differences in intelligence (22), to track normative brain network development in the first decade of life (using longitudinal data from the ABCD cohort) (23), and to discover case–control differences in brain network phenotypes across a range of rare genetic and other neurodevelopmental (24), psychiatric (25, 26), and neurological disorders (27).

Here, we used MSNs to integrate multiple macro- and microstructural MRI phenotypes, including features like cortical thickness and MT that have previously been linked separately to adolescent brain structural maturation (2), to measure developmental changes in individually estimated brain networks. We analyzed structural MRI scans in an accelerated longitudinal design (N = 291, age range 14 to 26 y; 51% female), stratified by age and balanced for sex per age stratum, with each participant scanned between one and three times at 6 to 18 mo intervals (*SI Appendix*, Fig. S1). From the six macro- and microstructural MRI metrics measured at each of 358 cortical areas, we estimated the morphometric similarity network for each of 469 individual scans. We used linear mixed effects models to estimate developmental change in MSN edge weights and nodal degree (a measure of hubness), and we compared these structural network results to functional networks constructed from 448 functional MRI scans in a large subset (N = 283) of the Neuroscience in Psychiatry Network (NSPN) sample. Recognizing the importance of replicability, we endeavored to reproduce key results from the NSPN cohort in an independent dataset, using N = 304 cross-sectional scans from subjects 14 to 21 y collected as part of the Human Connectome Project Development sample (HCP-D).

We hypothesized i) that there would be developmental changes in MSN phenotypes, e.g., some regional nodes might become more or less hub-like over the course of adolescence, and ii) that cytoarchitectonically distinct zones of the cortex, e.g., paralimbic cortex compared to the neocortex or isocortex, might have different trajectories of MSN development. We also predicted iii) that adolescent changes in structural networks should be related to concomitant changes in functional network organization.

## Results

### Analyzable Sample.

The final sample of morphometric feature data from the NSPN cohort after quality control consisted of 469 scans from 291 subjects in 358 regions. The fMRI sample included 448 scans from 283 subjects at 330 regions (*SI Appendix*, Table S1). We conducted each analysis on the largest possible dataset, thus analyses of brain structure were conducted on 291 subjects across 358 regions, whereas analyses of structure–function relationships were conducted on a subsample of 283 subjects across 330 regions (*SI Appendix*, Table S2). After quality control and age-matching, replication analyses were conducted using structural MRI data from N = 304 subjects of the HCP-D cohort.

### Adolescent Changes in Global and Regional MRI Metrics.

We first estimated adolescent changes at global and regional scales for six morphometric features: i) three macrostructural MRI metrics: cortical thickness, gray matter volume, and surface area; and ii) three microstructural metrics: magnetization transfer, fractional anisotropy, and mean diffusivity. We estimated the effect of age on these features using linear mixed effects (LME) models with a fixed effect of age, sex, and site, and a random effect of subject (Fig. 1*G*).

**Fig. 2.**
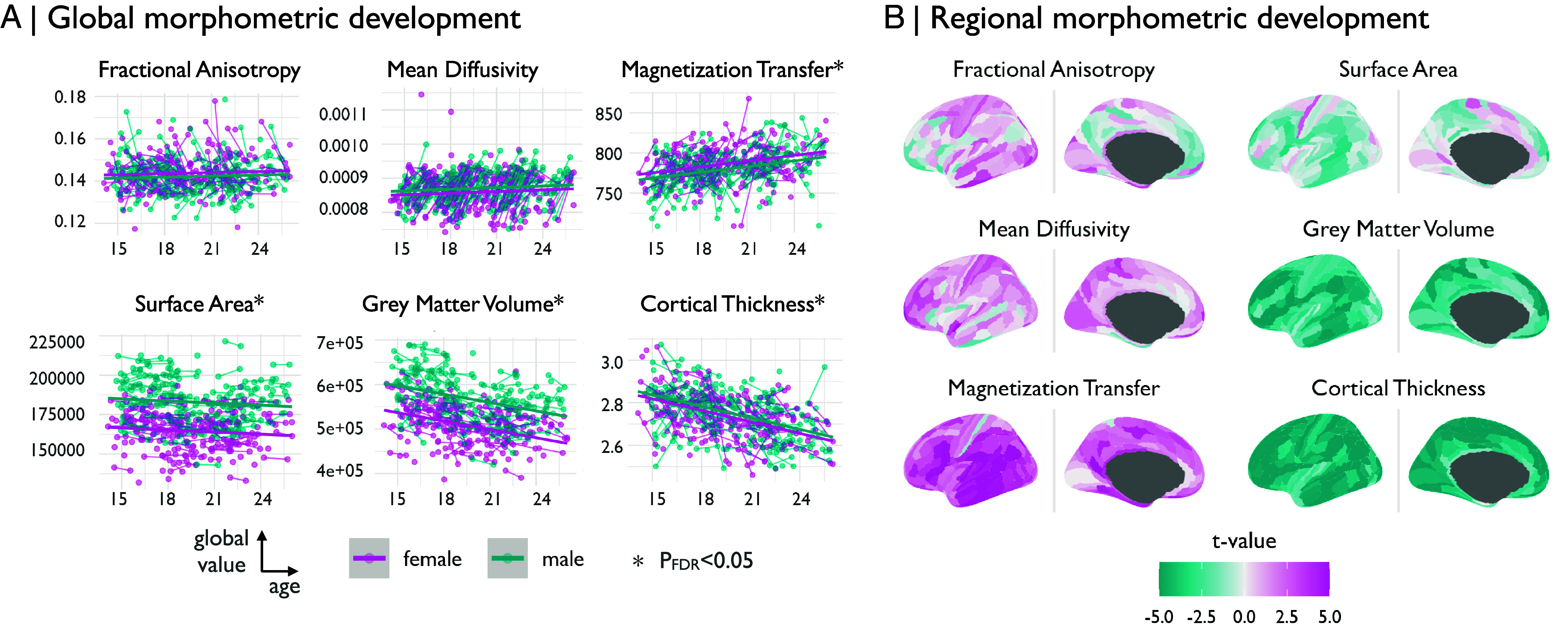
Adolescent changes in regional macrostructural and microstructural MRI metrics. We modeled the linear effect of age on six morphometric features. (*A*) Globally, the three macrostructural MRI metrics (GM, CT, and SA), all decreased over the course of adolescence ($P_{\;\text{FDR}}<0.05$ for each), while one of the three microstructural MRI metrics was significantly increased during adolescence (MT, $P_{\;\text{FDR}}<0.05$), but not MD or FA. (*B*) We modeled the linear effect of age on six morphometric features at each of 358 cortical areas to resolve the anatomical patterning of developmental changes in macro- and microstructural MRI metrics during adolescence. We generally observed increases (t>0) in microstructural features, and decreases (t<0) in macrostructural features.

Globally, we found that all macrostructural metrics significantly decreased during adolescence: SA, $t_{\;\text{age}}=-2.33,P_{\;\text{FDR}}<0.05$; GM, $t_{\;\text{age}}=-5.23,P_{\;\text{FDR}}<0.01$); and CT, $t_{\;\text{age}}=-7.29,P_{\;\text{FDR}}<0.01$. Of the microstructural metrics, MT significantly increased ($t_{\;\text{age}}=3.19;\;P_{\;\text{FDR}}<0.01$) while fractional anisotropy (FA) ($t_{\;\text{age}}=1.78$) and mean diffusivity (MD) ($t_{\;\text{age}}=-0.42$) showed no significant changes after correction for multiple comparisons (Fig. 2*A* and *SI Appendix*, Table 3). We also found that there were significant sex differences in one feature, GM, $t_{\;\text{sex}}=2.85,P_{\;\text{FDR}}<0.05$. For the subset of subjects with both baseline and 1-y follow-up scans, we also confirmed that these global trends were generally observed in the within-subject differences in global mean value of each MRI feature. The exception was FA, which was marginally increased on the group level, but on average, within-subject change was negative (for details, see *SI Appendix*, Fig. S4).

Mirroring the global developments, regionally, we found that macrostructural MRI metrics tended to decrease, and microstructural metrics tended to increase (Fig. 2*B* and *SI Appendix*, Fig. S5); We found that this effect was strongest for MT, where 289 regions significantly ($P_{\;\text{FDR}}<0.05$) increased in weighted degree over the course of adolescence, and in CT and GM, where 336 and 296 regions, respectively, significantly decreased (for a map of change relative to a feature’s global development see *SI Appendix*, Fig. S6). We found some variability between cytoarchitectonic zones of the cortex in terms of their age-related changes in each of the MRI metrics, e.g., all cortical zones had decreased macrostructural metrics but the magnitude of shrinkage was consistently less in the paralimbic cortex compared to neocortical zones (*SI Appendix*, Fig. S7 and Table S4).

### Adolescent Change in Morphometric Similarity.

We constructed MSNs from each participant’s set of T1 and DWI MRI scans, at each time-point, by estimating the Pearson correlation between all pairwise regional feature vectors comprising the six MRI metrics, resulting in a {358 × 358} symmetric morphometric similarity matrix or weighted, undirected MSN. The weighted degree, k, of each regional node in each MSN is a measure of its morphometric similarity with all other regions, and high-degree nodes or hubs are morphometrically similar to many other nodes in the brain.

**Fig. 3.**
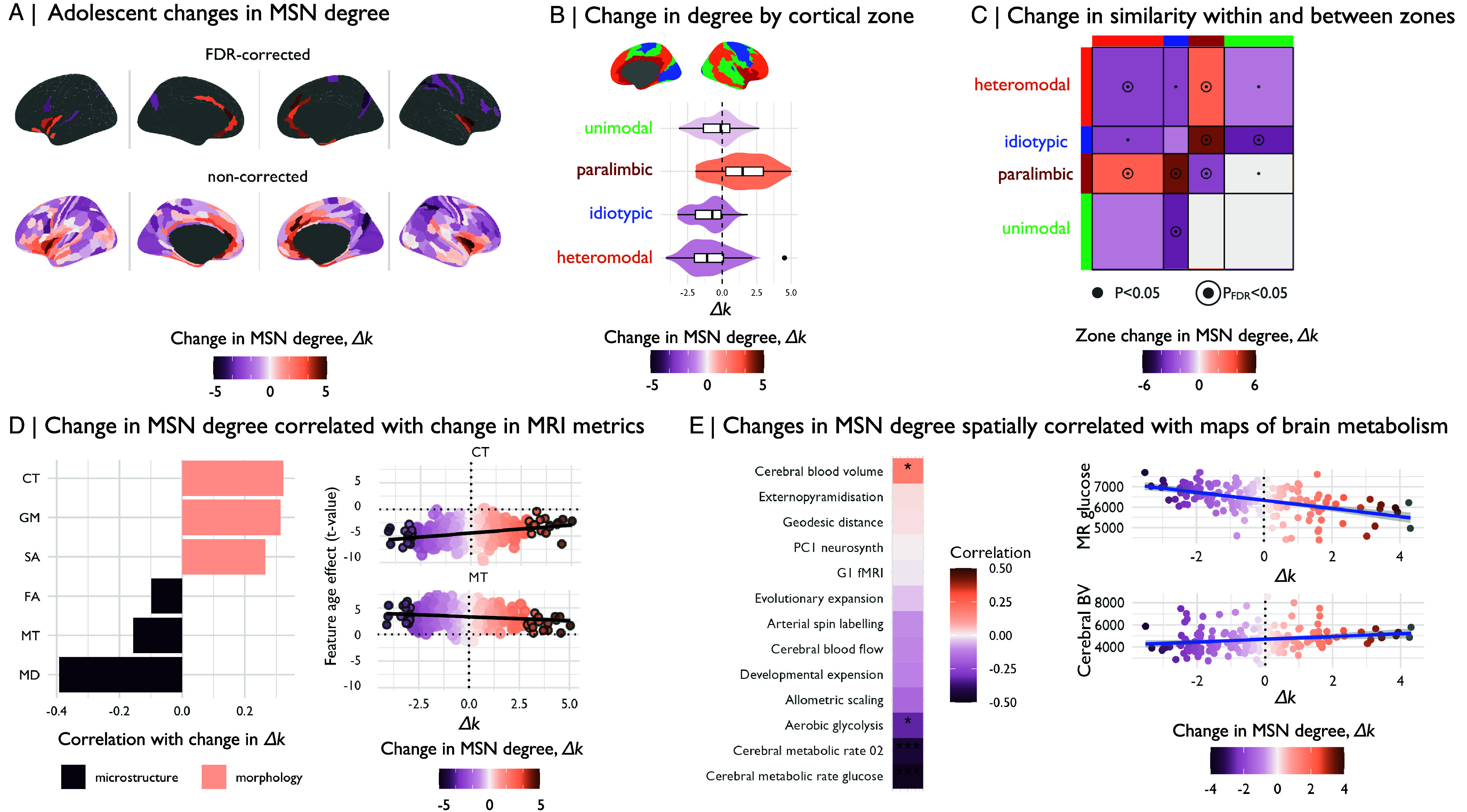
Adolescent change in degree of morphometric similarity, Δk. We estimated morphometric similarity networks for each subject by correlating the standardized morphometric feature vectors for each possible pair of regions. (*A*) We estimated linear changes in morphometric similarity with age, Δk, at each region and found that morphometric similarity decreased in neocortical (frontal, occipital) regions, and increased in medial and temporal cortical regions. These changes were significant after correction for multiple comparisons in 33 regions. (*B*) We estimated the mean effect of age on all regions within each of the Mesulam cytoarchitectonic zones (28, 29) and found that morphometric similarity increased in the paralimbic cortex and decreased in all other zones. (*C*) We also found that within-network similarity decreased for all zones, marginally in unimodal and idiotypic zones and significantly after correction for multiple comparisons in heteromodal and paralimbic zones ($P_{\;\text{FDR}}<0.05$), while between-network connectivity increased between paralimbic and heteromodal, as well as idiotypic zones ($P_{\;\text{FDR}}<0.05$) and decreased otherwise. (*D*) Next, we assessed the correlation between adolescent effects on individual MRI features at each region and the adolescent effect on degree of morphometric similarity, or “hubness,” of each regional node in the cortical connectome. We found that MT and other microstructural MRI features were negatively correlated with adolescent change in MSN degree, i.e., cortical myelination increased in areas that become more morphometrically dissimilar, or less hub-like with Δk<0, during adolescence. Conversely, macrostructural MRI features were positively correlated with adolescent change in MSN degree, i.e., cortical thickness, volume, and surface area all decreased in regions that became less hub-like during adolescence. Here, we highlight regions that are significant ($P_{\;\text{FDR}}<0.05$) Δk with gray outlines. (*E*) We estimated the correlation between the age effect on morphometric similarity and several prior maps of brain organization. We found a negative correlation between the effects of age on MSN nodal degree and several brain maps of metabolic rates, meaning that regions that showed decreases in degree of morphometric similarity tended to have increased metabolic rates. Conversely, the positive correlation between the age effect on MSN nodal degree and a map of cerebral blood volume means that regions that had decreased morphometric similarity over the course of adolescence had lower cerebral blood volume.

Because we constructed a MSN model of the connectome for each scanning session completed by each participant, we could estimate developmental changes in MSN parameters using the same LME as previously used for analysis of age-related change in global and regional MRI metrics (Fig. 1*G*). We found that weighted degree generally decreased with age, i.e., Δk<0, in heteromodal and other neocortical zones, meaning that these areas became more morphometrically dissimilar from the rest of the brain; whereas degree increased with age, i.e., Δk<0, in paralimbic areas such as the insula and cingulate cortex, meaning they became more morphometrically similar to the rest of the brain (Fig. 3*A*).

We did not find evidence for widespread sex differences in morphometric similarity during adolescence (*SI Appendix*, Fig. S8), and while some regions showed significant effects of scanning site, these regions did not overlap extensively with regions that showed significant developmental changes in morphometric similarity (*SI Appendix*, Fig. S9), the effects of site and age were not correlated (ρ=−0.07,P=0.16). To address the potential impact of site-specific factors on internal replicability of our results, we split the sample by site into three internal replication datasets (347 scans acquired at Wolfson Brain Imaging Center, 98 at University College London, and 33 at the MRC Cognition and Brain Sciences Unit). Despite the differences in site-specific sample size, we found a high spatial correspondence between our principal results on the whole NSPN cohort and the site-specific results derived from each of the three subcohorts (*SI Appendix*, Fig. S10). 33 regional MSN nodes, primarily located in transmodal areas of the paralimbic (N = 18) and heteromodal (N = 10) cortex, had significant changes in weighted degree after correction for multiple comparisons ($P_{\;\text{FDR}}<0.05$; see *SI Appendix*, Table 5 and Fig. 3*A*, *Top*). We further confirmed that the directionality of significant age effects on morphometric similarity degree (k) was robust: i) to sample size, using an ablation analysis which left out iteratively larger fractions of the data; and ii) to composition of the sample, using a permutation approach that resampled subjects with replacement (*SI Appendix*, Fig. S11). Last, we wanted to confirm that these between-subject, age-related changes in MSN degree were related to the developmental process of within-subject change in degree of similarity. However, we could not estimate within-subject linear rate of change as a random effect, because this would require at least three repeated measures per participant. Instead, we simply estimated the difference in MSN degree at baseline and 1-y follow-up at each cortical area for each of N = 172 participants and found that this measure of within-subject longitudinal change was significantly positively correlated with between-subject age-related changes in MSN degree (Δk; $\rho =0.41,P_{\;\text{spin}}<0.001$; see *SI Appendix*, Fig. 12).

To test our hypothesis that development of morphometric similarity should be conditioned by the cytoarchitectonic features of the cortex, we estimated the mean effect of age on weighted degree of all regions within each of four known cytoarchitectonic zones of the cortex (28, 29) (Fig. 3*B*). We found that isocortical or neocortical zones (idiotypic, unimodal, and heteromodal) had decreased MSN degree, i.e. Δk<0, whereas paralimbic cortical areas had increased degree, i.e. Δk>0 (Fig. 3*B* and *SI Appendix*, Table S6; for a breakdown of results by predefined functional subnetworks (30) and further cytoarchitectonic classes (31) refer to *SI Appendix*, Fig. S13). We further observed overall decreasing within-zone similarity, whereas between-zone similarity was specifically and significantly increased between the paralimbic and idiotypic cortex, as well as between the paralimbic and heteromodal cortex (Fig. 3*C*). Moreover, we estimated changes in the connectivity between each individual cytoarchitectonic zone and the rest of the brain (*SI Appendix*, Fig. S15*A*) and observed zone-specific patterns of reorganization in connectivity to the rest of the brain (*SI Appendix*, Fig. S11*B*).

Last, we endeavored to replicate our main findings from the NSPN cohort in an independent cohort. There was no other MRI dataset available to us that was directly comparable to NSPN in terms of providing repeated measures on MT. We instead used the HCP-D cohort (32) as an approximate replication sample, which provides measures of the T1w/T2w ratio as an alternative proxy of intracortical myelination (33). We found consistent age-related decreases in cortical thickness, surface area, and volume, and age-related increases in MT or T1w/T2w, across both HCP-D and NSPN cohorts ($P_{\;\text{spin}}<0.05$ for all correlations between original and replication maps). We note that one metric, fractional anisotropy, showed a diverging trend in the replication compared to the original sample ($P_{\;\text{spin}}=0.12$): FA showed no significant change over the course of adolescence in the NSPN cohort, but significantly decreased with age in the HCP-D cohort ($t=-2.5,P_{\;\text{FDR}}<0.05$). Most importantly, we found consistent results between cohorts in terms of age-related changes in degree of morphometric similarity, Δk, across different cytoarchitectonic classes of the cortex ($\rho =0.5,P_{\;\text{spin}}<0.001$, *SI Appendix*, Figs. S16 and S17). In MSNs derived from HCP-D data, as in MSNs from NSPN data, cortical areas in the paralimbic zone became increasingly similar, whereas areas of the heteromodal association cortex became increasingly dissimilar, over the course of adolescence (see *SI Appendix*, *Text* for details).

In an effort to understand the contribution of each of the six individual morphometric features to the adolescent change in morphometric similarity, we correlated the effect sizes of age (regional t-statistics) on the individual MRI phenotypes (Fig. 2*B*) with the age effects (regional t-statistic) on MSN weighted degree (Fig. 3*A*). We observed a divergent pattern: age-related changes in microstructural MRI markers were negatively correlated with adolescent changes in weighted degree (MD: $r=-0.4,P_{\;\text{spin}}<0.05$; MT: $r=-0.15,P_{\;\text{spin}}<0.05$; FA: r=−0.1), whereas macrostructural changes were positively correlated with adolescent changes in weighted degree (GM: $r=0.32,P_{\;\text{spin}}<0.05$; CT: $r=0.31,P_{\;\text{spin}}<0.05$; SA: $r=0.26,P_{\;\text{spin}}<0.05$) (Fig. 3*D* and *SI Appendix*, Fig. S18). This result indicates that regions of unimodal, heteromodal, and idiotypic cortex which became less morphometrically similar (or more morphometrically differentiated from the rest of the brain), as indexed by decreasing MSN degree during adolescence, tended also to become thinner and smaller, and more strongly myelinated (Fig. 3*D*). Thus the well-known adolescent processes of cortical thinning and increased myelination appeared to drive increasing morphometric dissimilarity or differentiation of neocortical nodes. Regions of the paralimbic cortex that had increased MSN degree over the course of adolescence showed a similar pattern of decreased macrostructural and increased microstructural metrics, but of smaller magnitude compared to isocortical areas, as indicated by t-values closer to zero (Fig. 3*D*).

### Biological and Psychological Context of Adolescent Changes in Anatomical Connectomes.

We were interested in contextualizing age-related changes in MSNs in relation to prior maps of transcriptional and functional gradients, evolutionary change, and metabolic requirements (34). We found that the whole brain map of adolescent change in weighted degree of each node was significantly negatively correlated with commensurate maps of aerobic glycosis ($r=-0.32;\;P_{\;\text{spin}}<0.05$) and the rates of oxygen ($r=-0.44;\;P_{\;\text{spin}}<0.001$) and glycolysis metabolism ($r=-0.48;\;P_{\;\text{spin}}<0.001$). Thus association and other isocortical nodes that had decreased MSN degree during adolescence tended to have increased metabolic demands in adulthood (Fig. 3*E*). Conversely, we found a positive correlation with a map of cerebral blood volume ($r=0.19;\;P_{\;\text{spin}}<0.05$), meaning that paralimbic regions that had increased MSN degree during adolescence tended to have decreased cerebral blood volume (Fig. 3*E*).

We also explored the psychological relevance of age-related changes in morphometric similarity. We conducted automated meta-analytic referencing using the NeuroSynth database of task-related fMRI activation coordinates (35). This analysis revealed that isocortical regions that showed decreases in MSN degree (t<0) were typically activated by tasks related to visual processing and imagery, motor control, and working memory. Conversely, paralimbic regions that showed increases (t>0) in MSN degree were associated with self-evaluation of emotional content, nociception, and pain (*SI Appendix*, Fig. S14).

### Adolescent Development of Structure–Function Coupling.

**Fig. 4.**
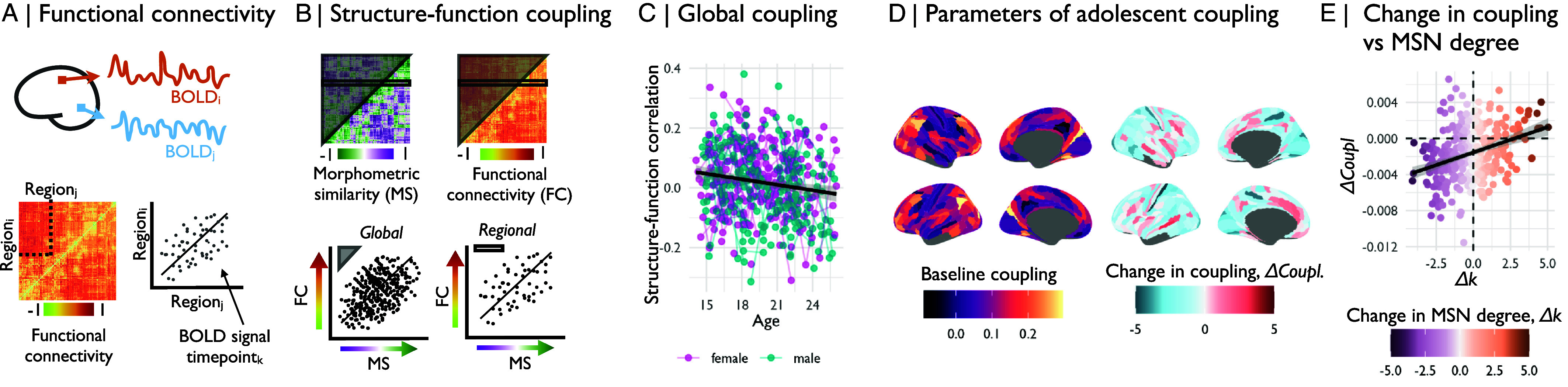
Adolescent development of structure–function coupling. (*A*) We derived a functional connectivity (FC) matrix, or functional connectome, for each scan by estimating the pairwise correlations between resting-state fMRI time series averaged over all voxels in each of all possible pairs of two regions defined by the parcellation template. (*B*) We estimated global structure–function coupling by correlating the ranked edgewise structural and functional connectivity vectors derived from each participant’s FC matrix and MSN, respectively; and regional structure–function coupling as the correlation between the ranked vector of a region’s edges derived from the FC matrix and the MSN, respectively. (*C*) We estimated the linear effect of age on global structure–function coupling and found that there was a significant decline in coupling over the course of adolescence (t=−5.04,P<0.001). We next estimated the linear effect of age on regional structure–function coupling using linear mixed effects models. From this analysis, we derived (*D*) a map of baseline structure–function coupling as the predicted coupling at age 14, and (*E*) a map of the rate of change in coupling, or the t-value of the effect of age on structure–function coupling. We found that 10 regions showed significant changes in structure–function coupling during adolescence, after correction for multiple comparisons ($P_{\;\text{FDR}}<0.05$; *SI Appendix*, Fig. S12*A*). (*E*) We found that the age effect on morphometric similarity was significantly positively correlated with the rate of change in coupling ($r=0.36,P_{\;\text{spin}}<0.01$). Thus, isocortical regions that become more morphometrically dissimilar (structurally differentiated) tended to have decreased strength of structure–function coupling over the course of adolescence.

We hypothesized that adolescent changes in brain structure, measured as increases or decreases of morphometric similarity, might change the strength of coupling between structural and functional brain networks (Fig. 4*A*). To test this hypothesis, we first estimated global structure–function coupling as the correlation between the ranked elements of the functional connectivity matrix and the morphometric similarity matrix for each subject, at each time-point (Fig. 4*B*). We modeled the linear effect of age on structure–function coupling using the same LME model as previously used for global, local, and MSN metrics. We found that global structure–function coupling decreased over the course of adolescence (t=−5.04,P<0.001; Fig. 4*C*), indicating a decoupling of functional connectivity from morphometric similarity.

We also tested this hypothesis regionally, estimating the correlations between the ranked elements of each row of the functional connectivity and morphometric similarity matrices (Fig. 4*B*), and then using the same LME to estimate age effects on regional structure–function coupling. From this analysis, we derived a map of baseline coupling, or the predicted coupling at age 14 y (Fig. 4*D*, *Left*), as well as a map of adolescent changes in regional coupling (Fig. 4*D*, *Right*). Baseline coupling was high in most isocortical areas, but lower in paralimbic areas (Fig. 4*D* and *SI Appendix*, Fig. S19*A* and Table S7). Coupling decreased most strongly in isocortical regions, and decreased less or increased slightly in paralimbic cortical regions (Fig. 4*E* and *SI Appendix*, Fig. S19*B* and Table S7). It is notable that the majority of regions decreased in coupling over the course of adolescence (blue in Fig. 4*D*). We also found that structure–function coupling at baseline and the rate of change in coupling were negatively correlated ($r=-0.35;\;P_{\;\text{spin}}<0.001$; *SI Appendix*, Fig. S20*C*), thus regions that were more strongly coupled at baseline tended to have greater decreases in coupling over the course of adolescence.

We further investigated how baseline regional structure–function decoupling and its adolescent changes were related to baseline and adolescent changes in morphometric similarity. We found that baseline MSN degree at age 14 y (*SI Appendix*, Fig. S21) was weakly correlated with baseline structure–function coupling ($r=0.15,P_{\;\text{spin}}<0.05$; *SI Appendix*, Fig. S20*B*), such that regional hubs at baseline had stronger structure–function coupling. There was also significant positive correlation between adolescent changes in MSN degree and structure–function coupling ($r=0.36,P_{\;\text{spin}}<0.01$; Fig. 4*F*), meaning that isocortical regions which became more dissimilar from the rest of the brain during adolescence had weaker structure–function coupling, whereas paralimbic regions which became more morphometrically similar had stronger coupling (Fig. 4*F* and *SI Appendix*, Fig. S20).

### Codevelopment of Structural and Functional Network Changes.

**Fig. 5.**
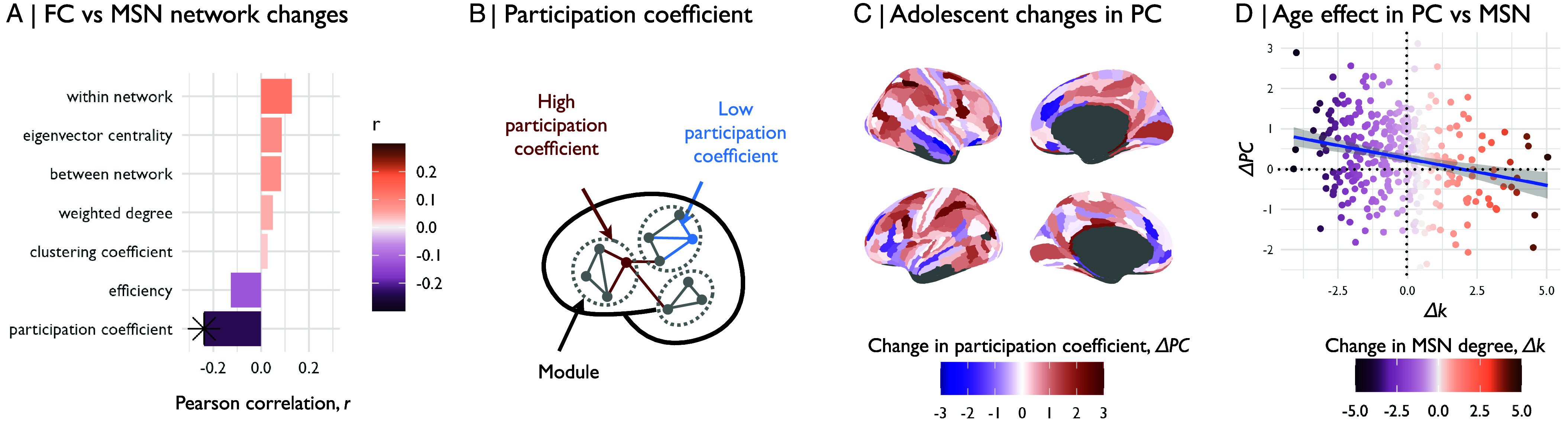
Morphometric dissimilarity was associated with functional participation. (*A*) We estimated age-related changes in multiple network metrics of regional nodes in fMRI connectomes over the course of adolescence. We then correlated the adolescence-related changes in these fMRI network phenotypes with changes in morphometric similarity. We found that only the participation coefficient (PC), a measure of intermodular connectivity, showed adolescent changes that were significantly associated with adolescent changes in MSN degree. (*B*) We then estimated the regional participation coefficient (PC) at each node for each connectome. PC is measured as the ratio between a node’s intramodular degree (edges connecting to other nodes in the same module) and its intermodular degree (edges connecting to nodes in other modules). (*C*) We estimated the linear effect of age on PC. Functional participation increased over the course of adolescence in association cortical regions and decreased in primary motor and sensory regions, as well as medial prefrontal regions. (*D*) We estimated Spearman’s correlation between regional age-related changes in morphometric similarity and regional age-related changes in functional participation coefficient. We found that regions that became more morphometrically dissimilar over the course of adolescence tended to increase in their functional participation.

In order to further understand how adolescent changes in structural brain networks relate to concomitant changes in brain functional networks, we estimated age-related changes in multiple network metrics (Fig. 5*A*). We did not find significant associations between adolescent changes in morphometric similarity and adolescent changes in weighted degree of functional connectivity ($r=-0.05,P_{\;\text{spin}}=0.4$; *SI Appendix*, Fig. S22 and Fig. 5*A*) or multiple other network metrics, including within- and between-network connectivity, eigenvector centrality, clustering coefficient, and efficiency (Fig. 5*A*). However, motivated by prior work (9), we were particularly interested to assess whether developmental changes in morphometric dissimilarity were associated with changes in participation coefficient, a measure of the topological diversity of functional connectivity between modules (Fig. 5*B*). Regions with a high participation coefficient have a relatively high proportion of intermodular connections to nodes in other modules, thus they may have the capacity to integrate information across multiple subgraphs or modules of the whole brain connectome and have been designated as “connector hubs.” Conversely, regions with a low participation coefficient have more locally segregated connectivity within their respective modules and have previously been designated “provincial hubs” because of their important role in communication between modules (36). We found that adolescent increases in regional participation coefficient were largely located in isocortical regions and decreases were more concentrated in paralimbic regions, as well as medial prefrontal regions (Fig. 5*C*). Further, we found that adolescent changes in MSN degree were correlated with adolescent changes in PC ($r=-0.24,P_{\;\text{spin}}<0.01$, Fig. 5*A*, *Bottom*), such that isocortical regions that became more morphometrically dissimilar over the course of adolescence had increased PC over the same period (Fig. 5*D* and *SI Appendix*, Fig. S23 and Table S8), whereas paralimbic regions that became more morphometrically similar had decreased PC during adolescence. We thus established that increases in morphometric dissimilarity, or structural differentiation from the rest of the brain, were associated with increasing diversity of functional connectivity, measured as a relative strengthening of intermodular connectivity, potentially representing an increased ability to integrate information across multiple, structurally differentiated modules.

## Discussion

We set out to investigate the hypothesis that there are developmental changes in human brain structural networks during adolescence, and that cortical areas may become more or less hub-like in the connectome depending on their maturing cytoarchitectonic differentiation from the rest of the cortex. We used morphometric similarity analysis of multiple MRI metrics to measure brain anatomical networks from repeated structural MRI assessments of a large cohort of healthy young people. We found that there were indeed significant developmental changes in the hubness of cortical areas during adolescence, and that the regional trajectories of adolescent change in MSN hubness or degree, Δk, were remarkably distinct between paralimbic and all other (isocortical) zones of the cortex.

According to the cytoarchitectonic scheme defined by Mesulam (28, 29), the isocortex or neocortex is defined by six cortical layers, it encompasses the majority of the human cortex, and it can be subdivided into three zones of the idiotypic, unimodal, and heteromodal cortex. We found that almost all such isocortical areas became less similar or more dissimilar from the rest of the cortex during adolescence, and therefore less hub-like in morphometric similarity networks. The most likely interpretation of this process of “dehubification” is that each of these areas is becoming more structurally differentiated from the rest of the brain, more unique in its cytoarchitectonic or myeloarchitectonic organization. We found that isocortical regions with decreasing hubness typically had more rapidly shrinking cortical thickness, volume, and surface area, and greater increases in myelination indexed by MT. Coupled changes in cortical shrinkage and cortical myelination have been well replicated in prior neurodevelopmental MRI studies of childhood and adolescence (2, 5) and can be interpreted as a process of pruning connections at a cellular level, and consolidating those connections that survive by myelination. It therefore seems likely that the increasing dissimilarity of isocortical nodes in morphometric similarity networks reflects their increasingly selected and distinctive profile of (myelinated) anatomical connections to the rest of the brain.

The paralimbic zone is defined by having less than six cortical layers and lacking a well-defined layer four of granule cells. It represents a minority of the total cortex, including insular, orbito-frontal, temporal polar, and cingulate cortices. Cytoarchitectonically and connectionally, the paralimbic zone is regarded as transitional or intermediate between the most phylogenetically primitive, 3-layered regions of allocortex, e.g., hippocampus or pyriform cortex, and the 6-layered neocortex or isocortex. We found that almost all paralimbic areas became more morphometrically similar, or more hub-like, over the course of adolescence. There are at least two plausible interpretations of this result: local (absolute) or contextual (relative) change in cortical structure. Locally, it could be that the paralimbic cortex becomes “less primitive” or cytoarchitectonically more similar compared to the isocortex during adolescence. Across the insula of the primate brain, for example, there is an antero-posterior gradient of cortical architectonics from the agranular, periallocortical organization of anterior (rostral) insula, continuous with the 3-layered pyriform cortex, to the dysgranular, proisocortical organization of posterior (caudal) insula, continuous with 6-layered heteromodal and unimodal association areas of the temporal and parietal cortex (37). This cytoarchitectonic gradient has evolved phylogenetically from reptiles to primates, and it is conceivable that this process might be recapitulated ontogenetically, with an increasing proportion of the insula becoming organized more like isocortex and less like allocortex over the course of development. This would be consistent with our observations of greater morphometric similarity between isocortical (idiotypic and heteromodal) and paralimbic zones, and therefore higher degree of paralimbic cortical nodes, over the course of adolescent MSN development. An alternative interpretation is that the paralimbic cortex becomes relatively more similar to the isocortex, as the isocortical areas become relatively more dissimilar to (differentiated from) each other. In other words, there may be no local cytoarchitectonic maturation of progressively more isocortical lamination but paralimbic areas could nonetheless appear to become more similar “on average” across the brain as the corollary of isocortical or neocortical differentiation associated with adolescent shrinkage and myelination. Further studies linking MRI metrics and morphometric similarity to underlying cellular changes in development of the mammalian cortex will be required for definitive mechanistic resolution of the increasing hubness of the paralimbic cortex, as well as to confirm the pruning-and-myelination mechanism proposed to account for decreasing hubness of the isocortex during adolescence. Indeed, prior work in children has linked morphometric dissimilarity between isocortical and paralimbic zones to better cognitive performance, suggesting that structural differentiation is a prerequisite for healthy development in the preadolescent period, a finding which supports the relevance of understanding changes in morphometric similarity during development (23).

We proceeded to investigate the consequences for brain functional connectivity of this cytoarchitectonically aligned divergence in structural network development, and made two interesting observations. First, we found that morphometric similarity and functional connectivity, measured in the same scanning session, were significantly but modestly coupled at baseline (r≤0.25 at 14 y) and the strength of structure–function coupling globally declined over the course of adolescence, as previously reported (38). However, at a regional level, there was again some evidence for cytoarchitectonically aligned divergence in developmental trajectories. Isocortical areas tended to have reduced structure–function coupling over the course of adolescence compared to paralimbic areas. And adolescent change in structure–function coupling at each region was positively correlated with the adolescent change in MSN degree that was previously shown to be cytoarchitectonically aligned. Second, we found that the participation coefficients for each regional node in the fMRI connectomes were also developmentally divergent on cytoarchitectonic lines: most isocortical areas had increased participation and most paralimbic areas had decreased participation; and adolescent change in participation was positively correlated with the cytoarchitectonically aligned adolescent change in MSN degree. Collectively these results suggest that as isocortical areas become less hub-like or more structurally differentiated their pattern of functional interactions with other cortical areas becomes less constrained or more diverse. Morphometric similarity is a proxy for (often reciprocal) monosynaptic connectivity between areas of same cytoarchitectonic class, whereas functional connectivity is often regarded as a proxy for polysynaptic connectivity. Low strength of structure–function coupling and high participation means that mature isocortical areas can interact functionally with other areas even if they are cytoarchitectonically dissimilar or affiliated to different modules of the fMRI connectome, possibly relying more on polysynaptic (indirect) axonal connections or circuit-level modulation of neuronal activity (9). Given prior theories on the importance of network integration and segregation to different aspects of cognition (9), this developmental shift of heteromodal and other isocortical nodes to increasing diversity of functional connectivity and increasing differentiation of cortical structure could be relevant to the emergence of a wide range of domain-general or “higher-order” cognitive skills which depend on integrative network properties (39). The opposite trend demonstrated by some (mostly paralimbic) areas—toward increased structure–function coupling and decreased participation—convergently suggests consolidation of intramodular interactions between morphometrically similar areas specialized for a specific function, e.g., interoception, that emerges during adolescence. Further work will be needed to test these predictions of the cognitive consequences of cytoarchitectonically aligned changes in MSN and fMRI network connectivity.

There are some notable methodological issues. First of all, the test–retest reliability of MSNs has not previously been assessed. Here, we estimated the longitudinal (between-visit or within-subject) rank stability of global and regional MSN degree as a proxy for test–retest reliability and found generally high levels of rank stability. Rank of regional MSN degree was significantly positively correlated between baseline and follow-up scans in 241 regions (*SI Appendix*, *Text* and Fig. S24). We found that 21 of the 33 regions that showed significant changes in MSN degree also had high rank stability, indicating that the majority of developmental changes in MSN degree are not in less reliable regions (*SI Appendix*, Fig. S24). Future work will have to assess test–retest reliability of MSNs more directly. Only a minority of cortical areas demonstrated significantly nonzero changes in similarity during adolescence yet most of our analysis has included data from all cortical areas, e.g., to investigate differences in development of similarity between cytoarchitectonic classes or cortical types. In doing so, we assumed that developmental change in similarity was widespread throughout the brain but our statistical power to demonstrate significant change at a regional level was constrained by the number of participants, the number of scans per participant, the nominal degrees of freedom for estimation of similarity in each scan, and the multiple comparisons correction needed to control FDR<5% for all 358 cortical regions tested. We expect that future studies of brain similarity development will achieve greater statistical power by addressing some or all of these constraints. A notable strength of this analysis was the generally convergent results from two independent datasets. However, it is noteworthy that not all individual analyses were replicated. For example, we observed discrepancies in the global development of FA between the NSPN and HCP-D (replication) samples. A number of reasons may have contributed to this discrepancy, including differences in sample size and preprocessing pipelines between the two cohorts. Even taking this specific discrepancy into account, age-related trends in adolescent development of morphometric similarity were largely replicated between the two cohorts, which supports the robustness of our methods and these results. Further, to date, there is no consensus on how best to estimate the coupling between structural and functional connectivity, and various methods have been used to define both structural and functional networks (9, 38, 40), which may contribute to the lack of consistency in the overall pattern of previously reported results. Notably, structure has so far been defined from DWI networks (9) or graph theoretical properties of such networks (38). However, it is conceivable that new insights can be gained into how structure constrains function by employing different structural network modeling approaches, including more directly modeling relevant maturational processes of increasing myelination paired with cortical thinning, as is possible using morphometric similarity networks.

Overall, we conclude that adolescence is associated with an extensive developmental program of increasing structural differentiation and functional integration of isocortical zones, and increasing structural similarity and functional segregation of the paralimbic cortex.

## Materials and Methods

This study included data from an accelerated longitudinal study (41) of adolescents ages 14 to 26 y (51 % female) who were invited to undergo functional and structural neuroimaging assessments on at least two occasions: at baseline and at a 1-y follow-up assessment, with a subset of the sample invited to come in 6 mo after baseline for an additional scan (*SI Appendix*, Fig. S1). Participants provided informed written consent for each aspect of the study, and parental consent was obtained for those aged 14 to 15 y. The study was ethically approved by the National Research Ethics Service and conducted in accordance with U.K. National Health Service research governance standards (see *SI Appendix*, *Text* for further details).

### Structural MRI Acquisition and Preprocessing.

The MRI data were acquired using a multiparametric mapping sequence (42) at three sites, on three identical 3T Siemens MRI scanners (Magnetom TIM Trio, VB17 software version) with a standard 32-channel radio-frequency (RF) receive head coil and RF body coil for transmission. The anatomical, diffusion weighted, and functional imaging data were collected during the same session. The anatomical MRI data were acquired using a single-shot echo planar imaging sequence (63 gradient directions with b-value = 1,000 mm/s^2^ and 5 unweighted B0 images) was used to acquire a HARDI with the following scanning parameters: slice number = 70 consecutive; slice thickness = 2 mm; field of view = 192 × 192 mm; TE = 90 ms; TR = 8,700 ms; and voxel size = 2.0 mm isotropic.

We preprocessed the anatomical data using the recon-all command in Freesurfer v5.3.0 (43). In short, the pipeline included the following steps: nonuniformity correction, projection to Talairach space, intensity normalization, skull stripping, automatic tissue, and subcortical segmentation, and construction of smooth representations of the gray/white interface and the pial surface. Subsequently, the DWI volumes were aligned to the R1 image for each subject. Last, we parcellated the anatomical and DWI scans into 360 bilateral parcels, using the Human Connectome Project (HCP) parcellation atlas (44).

### fMRI Acquisition and Preprocessing.

The functional MRI data were acquired using a multiecho (ME) echo-planar imaging sequence with the following scanning parameters: TR = 2.42 s; GRAPPA with acceleration factor = 2; flip angle = 90°; matrix size = 64 × 64 × 34; field of view = 240 mm by 240 mm; in-plane resolution = 3.75 mm by 3.75 mm; slice thickness = 3.75 mm with 10% gap, with sequential acquisition of 34 oblique slices; bandwidth = 2,368 Hz/pixel; and TE = 13, 30.55, and 48.1 ms.

The preprocessing pipeline has been described in depth in prior publications (45, 46). Briefly, this included ME-ICA to remove non-BOLD components (47, 48); CSF regression using Analysis of Functional NeuroImages software [AFNI; (49)]; parcellation into 360 bilateral cortical regions using the HCP template (44); band-pass filtering (frequency range 0.025 to 0.111 Hz); removal of 30 dropout regions, defined by a low Z score of mean signal intensity in at least one participant (Z<−1.96); functional connectivity estimation using Pearson’s correlation between all possible combinations of regional timeseries; and Fisher’s r−Z transformation. Finally, to remove any residual effects of head motion on functional connectivity, we regressed each pairwise correlation between regions on the time-averaged head motion of each participant (mean framewise displacement). We retained the residuals of this regression, i.e., motion-corrected Z scores, as the estimates of functional connectivity for this analysis.

### Morphometric Feature Estimation and Quality Control.

We derived FreeSurfer’s standard morphometric features: CT, GM, SA. Previous work on this sample had indicated that MT adolescent changes with age were most pronounced at 70% cortical depth from the pial surface (2); thus regional MT values were estimated at that depth. Last, regional volumes for FA and MD were derived from the DWI scans.

We excluded 11 subjects due to outliers in at least one morphometric feature (MAD≥5; see *SI Appendix*, Fig. S2 and *Text* for further details). Two regions were excluded due to a local signal dropout, defined as MAD=0 in at least one morphometric feature across subjects, which led to the exclusion of two regions (*L_H, R_H*), such that the total number of regions analyzed henceforth was 358 (see *SI Appendix*, Fig. S3 and Text for further details).

### Modeling of Developmental Change in Morphometric Features.

We estimated linear age-related changes or development in six morphometric features at global scale, i.e., on average for each feature over all regions, and locally, for each feature f at each region (i=1...N=358), using linear mixed effects models, with a fixed effect of age, sex and site, and a random effect for the repeated measures on each participant, as follows:

[1] $$\begin{array}{cc} f_{i}∼1 & +\;\beta_{\;\text{age}}*age+\beta_{\;\text{sex}}*sex+\beta_{\;\text{site}}*site\\ & +\;\gamma_{\;\text{subject}}*\left(1|subject\right)+\epsilon , \end{array}$$

where $f_{i}$ refers to the morphometric feature value at region i, β refers to the coefficients for the fixed effects, $\gamma_{\;\text{subject}}$ refers to the coefficients for the random effect, and ϵ represents the residual error.

### Adolescent Changes in Morphometric Similarity.

We derived subject-specific structural connectomes, i.e. morphometric similarity networks. To this end, we standardized each morphometric feature within each subject using MAD (50). We then estimated morphometric similarity networks for each subject by calculating the Pearson correlation between their standardized feature vectors for each possible pair of regions. This resulted in a 358 × 358 symmetric matrix, indicative of morphometric similarity between cortical regions.

We first estimated regional morphometric similarity, or weighted degree k as the mean across a region’s edges. Then we estimated the linear effect of age on MSN weighted degree, using linear mixed effects models (Fig. 1*G*) with a fixed effect of age, sex, and site and random effect of subject, as follows:

[2] $$\begin{array}{cc} k_{i}∼1 & +\;\beta_{\;\text{age}}*age+\beta_{\;\text{sex}}*sex+\beta_{\;\text{site}}*site\\ & +\;\gamma_{\;\text{subject}}*\left(1|subject\right)+\epsilon , \end{array}$$

where $k_{i}$ refers to the morphometric similarity strength, or weighted degree, of regional node i, β refers to coefficients for the fixed effects, $\gamma_{\;\text{subject}}$ refers to the coefficients for random effects, and ϵ represents the residual error. From this model, we estimated the adolescent rate of change in morphometric similarity, or the age effect on weighted degree at each node of MSN, as the t-statistic of the age effect.

We tested for the robustness of the derived age-related changes in morphometric similarity using two sensitivity analyses: First, we estimated the correlation between age effects derived using an ablation analysis of ever-decreasing sample sizes, i.e. we randomly sampled decreasing numbers of subjects and re-estimated the effect of age on the smaller samples. Second, we derived CIs around the estimated age effects in a leave-N-out bootstrap analysis, where we randomly left out 10% of the sample in 1,000 permutations and re-estimated the age effects on morphometric similarity (see *SI Appendix*, Fig. 8 for details).

In order to decode the regional changes in morphometric similarity by cell type and functional modules, we averaged weighted degree over all regions within each cytoarchitectonic zone and functional network of cortical areas defined a priori by the respective reference brain atlas (28–31).

We then estimated the correlation between age-related changes (t-values) in individual morphometric features, estimated by Eq. **1**, and age-related changes (t-values) in morphometric similarity, estimated by Eq. **2**. Each analysis of spatial colocation or correlation between two cortical maps was reported with both the parametric P-value corresponding to the Pearson correlation (r), as well as a P-value derived from the more conservative “spin-test” permutation. Spatial autocorrelation of statistical brain maps can cause inflated estimates of the probability of spatial colocation or correlation between two maps (4, 51). The spin test procedure addresses this issue by conserving the spatial autocorrelation of the maps by randomly “spinning” or spherically rotating each map 1,000 times over the surface of the brain and calculating the spatial colocation statistic after each spin permutation.

We estimated the anatomical colocation of the map of age-related changes in morphometric similarity with various maps of cortical organization, including metabolic rates, blood volume, and functional hierarchy (34). To do this, we correlated the ranked map of age-related changes in morphometric similarity with each prior map, and then estimated the significance of the correlation while controlling for spatial autocorrelation using a spin-test (4).

We further assessed the psychological relevance of the map of age-related changes in morphometric similarity using Neurosynth, an automated meta-analytical tool (35). We generated a volumetric version of the regional map of adolescent changes in morphometric similarity (code available at https://github.com/LenaDorfschmidt/neurosynth_analysis) and uploaded it for automated comparison to the Neurosynth database (https://neurosynth.org) of task-related fMRI activation coordinates, which returned the correlation values of the map with a wide set of terms related to fMRI task activation experiments.

### Replication of Results in an Independent Dataset.

We determined the replicability of our main results in an independent dataset, the HCP-D sample (32), a cross-sectional cohort of children and adolescents aged 5 to 21 y, for which multimodal imaging data, including T1w and T2w images, as well as DWI data were acquired. For better comparison with the NSPN sample, we included only HCP-D subjects aged 14 and older (N = 334). Participants aged 18 y and older provided informed written consent, for younger participants consent was obtained from a legal guardian who additionally accompanied the minor to testing. See *SI Appendix* for details on dataset demographics and processing.

### Adolescent Changes in Structure–Function Coupling.

We estimated global structure–function coupling as the Spearman correlation between the upper triangle of each subjects’s structural (MSN) and functional connectivity (FC) networks at each timepoint (Fig. 4 *A* and *B*). Local structure–function coupling was estimated at each node as the Spearman correlation between the node’s edges in the structural and functional networks (Fig. 4 *A* and *B*).

Then, we estimated parameters of adolescent change in structure–function coupling. Specifically, we estimated the linear effect of age on regional structure–function coupling strength using linear mixed effects models, with a fixed effect of age, sex, and site, and a random effect of subject, as above for MSN regional strength (Eq. **2**). From this model, we proceeded to derive the local structure–function coupling at baseline (age 14) and the rate of change in coupling over the course of adolescence, as the t-values of the effect of age (see *SI Appendix*, *Text* for further details).

### Colocation with Adolescent Changes in Functional Diversity.

We assessed whether changes in morphometric similarity during adolescence were associated with changes in functional brain networks which might represent adolescent changes in cognition and behavior. We estimated multiple network metrics on the thresholded functional connectomes (Fig. 5*A*) and used linear mixed effects models as above to estimate the linear effect of age on each network metric (see *SI Appendix*, *Text* for further details).

## Supplementary Material

Appendix 01 (PDF)

Dataset S01 (DOCX)

## Data Availability

The NSPN raw data is publicly available at https://nspn.org.uk (52) and the processed data can be downloaded from https://doi.org/10.5281/zenodo.11175459 (53). The HCP-D data are publicly available at https://www.humanconnectome.org (54). The code can be accessed at https://github.com/LenaDorfschmidt/morphometric-similarity-adolescence (55).
