# Supplementary material for: Human adolescent brain similarity development is different for paralimbic versus neocortical zones
Source: Proc Natl Acad Sci U S A. 2024 Aug 9;121(33):e2314074121. doi: 10.1073/pnas.2314074121 (PMC11331068; doi:10.1073/pnas.2314074121)
Supplement: Supplementary file 2 — Dataset S01 (DOCX) [file pnas.2314074121.sd01.docx]

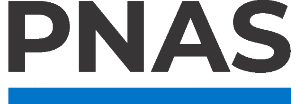


### ****Neuroscience in Psychiatry Network Consortium Authors****

#### ****Principal investigators****

Edward Bullmore (CI from 01/01/2017) ^1,2,3^

Raymond Dolan ^4,5^

Ian Goodyer (CI until 01/01/2017) ^1^

Peter Fonagy ^6^

Peter Jones ^1^

#### ****NSPN (funded) staff****

Michael Moutoussis ^4,5^

Tobias Hauser ^4,5^

Sharon Neufeld ^1^

Rafael Romero-Garcia ^1,2^

Michelle St Clair ^1^

Petra Vértes ^1,2^

Kirstie Whitaker ^1,2^

Becky Inkster ^1^

Gita Prabhu ^4,5^

Cinly Ooi ^1^

Umar Toseeb ^1^

Barry Widmer ^1^

Junaid Bhatti ^1^

Laura Villis ^1^

Ayesha Alrumaithi ^1^

Sarah Birt ^1^

Aislinn Bowler ^5^

Kalia Cleridou ^5^

Hina Dadabhoy ^5^

Emma Davies ^1^

Ashlyn Firkins ^1^

Sian Granville ^5^

Elizabeth Harding ^5^

Alexandra Hopkins ^4,5^

Daniel Isaacs ^5^

Janchai King ^5^

Danae Kokorikou ^5,6^

Christina Maurice ^1^

Cleo McIntosh ^1^

Jessica Memarzia ^1^

Harriet Mills ^5^

Ciara O’Donnell ^1^

Sara Pantaleone ^5^

Jenny Scott ^1^

Beatrice Kiddle^1^

Ela Polek^1^

#### ****Affiliated scientists****

Pasco Fearon ^6^

John Suckling ^1^

Anne-Laura van Harmelen ^1^

Rogier Kievit ^4,7^

Sam Chamberlain^1^

Richard A.I. Bethlehem ^1^

Affiliations were correct at time of compiling the list

1 Department of Psychiatry, University of Cambridge, United Kingdom

2 Behavioural and Clinical Neuroscience Institute, University of Cambridge, United Kingdom

3 ImmunoPsychiatry, GlaxoSmithKline Research and Development, United Kingdom

4 Max Planck University College London Centre for Computational Psychiatry and Ageing Research,

University College London, UK

5 Wellcome Centre for Human Neuroimaging, University College London, United Kingdom

6 Research Department of Clinical, Educational and Health Psychology, University College London,

United Kingdom

7 Medical Research Council Cognition and Brain Sciences Unit, University of Cambridge, United Kingdom
